# TET-dependent regulation of retrotransposable elements in mouse embryonic stem cells

**DOI:** 10.1186/s13059-016-1096-8

**Published:** 2016-11-18

**Authors:** Lorenzo de la Rica, Özgen Deniz, Kevin C. L. Cheng, Christopher D. Todd, Cristina Cruz, Jonathan Houseley, Miguel R. Branco

**Affiliations:** 1 Blizard Institute, Barts and The London School of Medicine and Dentistry, QMUL, London, E1 2AT UK; 2 Epigenetics Programme, Babraham Institute, Cambridge, CB22 3AT UK

**Keywords:** Embryonic stem cells, Retrotransposons, LINE-1, DNA methylation, Hydroxymethylation, Ten-eleven translocation enzymes, Enhancers

## Abstract

**Background:**

Ten-eleven translocation (TET) enzymes oxidise DNA methylation as part of an active demethylation pathway. Despite extensive research into the role of TETs in genome regulation, little is known about their effect on transposable elements (TEs), which make up nearly half of the mouse and human genomes. Epigenetic mechanisms controlling TEs have the potential to affect their mobility and to drive the co-adoption of TEs for the benefit of the host.

**Results:**

We performed a detailed investigation of the role of TET enzymes in the regulation of TEs in mouse embryonic stem cells (ESCs). We find that TET1 and TET2 bind multiple TE classes that harbour a variety of epigenetic signatures indicative of different functional roles. TETs co-bind with pluripotency factors to enhancer-like TEs that interact with highly expressed genes in ESCs whose expression is partly maintained by TET2-mediated DNA demethylation. TETs and 5-hydroxymethylcytosine (5hmC) are also strongly enriched at the 5′ UTR of full-length, evolutionarily young LINE-1 elements, a pattern that is conserved in human ESCs. TETs drive LINE-1 demethylation, but surprisingly, LINE-1s are kept repressed through additional TET-dependent activities. We find that the SIN3A co-repressive complex binds to LINE-1s, ensuring their repression in a TET1-dependent manner.

**Conclusions:**

Our data implicate TET enzymes in the evolutionary dynamics of TEs, both in the context of exaptation processes and of retrotransposition control. The dual role of TET action on LINE-1s may reflect the evolutionary battle between TEs and the host.

**Electronic supplementary material:**

The online version of this article (doi:10.1186/s13059-016-1096-8) contains supplementary material, which is available to authorized users.

## Background

Transposable elements (TEs) are mobile genetic elements that are present in organisms from all kingdoms of life and account for nearly half of the human and murine genomes. Retrotransposons, which act through an RNA intermediate, are recognised as major evolutionary contributors to genome structure and organisation. Namely, retrotransposons bear enormous potential for affecting gene expression at multiple levels, from creating novel transcription factor (TF) binding sites to regulating post-transcriptional processes [1, 2]. The co-adoption, or exaptation, of TEs by the host genome is often associated with the creation of species-specific cis-acting elements that help to shape gene regulatory networks [1, 3]. In embryonic stem cells (ESCs), long-terminal repeat (LTR) TEs (also termed endogenous retroviruses; ERVs) have made a substantial contribution to the distribution of binding sites for pluripotency factors across both human and mouse genomes [4, 5], and the expression of HERVH TEs is required to safeguard the identity of human ESCs [6, 7].

On the other hand, active TEs such as LINE-1 (or L1) elements carry substantial mutagenic potential, as evidenced by numerous examples of disease-causing retrotransposition events [8]. Somatic retrotransposition of L1s has also been linked to disease, especially in the context of cancer [9, 10]. Mobility restriction of these elements is therefore of paramount importance to maintain genome stability, especially during the crucial developmental windows of germline establishment and early embryogenesis. Notably, both human ESCs and induced pluripotent stem cells (iPSCs) support L1 mobility [11] and de novo retrotransposition events from endogenous TEs have been detected in clonally derived iPSCs [12], with important implications for regenerative medicine strategies.

During early embryogenesis, TEs are kept under control by multiple epigenetic mechanisms such as DNA methylation, histone modifications and small RNA-mediated mechanisms [13]. The large and rapidly evolving family of Krüppel-associated box domain-containing zinc finger proteins (KRAB-ZFPs) play a major role in ERV silencing by recruiting the cofactor KRAB-associated protein 1 (KAP1), which acts as a docking protein for heterochromatin-forming activities, including the H3K9 methyltransferase SETDB1 [14, 15]. Interestingly, KRAB-ZFPs have also been implicated in the silencing of old L1 elements, acting as major drivers of L1 evolutionary dynamics [16, 17]. In contrast, Piwi-interacting RNAs (piRNAs) are important for silencing of young, active L1s in humans iPSCs [18]. DNA methyltransferases, which can act downstream of piRNA action, also play a crucial role in the repression of young L1 families in both mouse and human ESCs [15, 16, 19]. However, DNA methylation (5-methylcytosine; 5mC) undergoes genome-wide erasure during preimplantation development [20], creating a potential window of opportunity for TE expression. While the primary mechanism underlying DNA demethylation in this period is replication-coupled dilution of 5mC [21, 22], an active mechanism dependent on ten-eleven translocation (TET) enzymes has also been demonstrated for specific loci [23–25]. TETs catalyse the iterative oxidation of 5mC to 5-hydroxymethylcytosine (5hmC), 5-formylcytosine (5fC) and 5-carboxylcytosine (5caC), with the latter two bases being substrates for TDG-dependent repair mechanisms that reintroduce unmodified cytosines into the genome [26]. TET enzymes can also modulate transcription in a non-catalytic way by recruiting other chromatin regulators, such as OGT (O-linked β-d-N-acetylglucosamine (O-GlcNAc) transferase) [27], the SIN3 co-repressor complex [28] and the Polycomb repressive complex 2 (PRC2) [29]. Unlike TET-mediated DNA demethylation, the interaction with these proteins can have repressive outcomes, underlying a dual nature of TET action in ESCs [28, 30].

We have previously shown that L1 elements are enriched in 5hmC in mouse ESCs [31, 32], raising the possibility that TET enzymes regulate their transcription. However, a wider and functional investigation of the role of TET enzymes at TEs is missing. Here we asked whether TET enzymes regulate the action of TEs, both as exapted regulatory elements and as mutagenic mobile elements. We found that TETs bind to multiple TE families, including TE-derived ESC-specific enhancers, which are kept hypomethylated in part by the action of TET2, thus helping to maintain the expression of associated genes. We also show that TETs demethylate young L1 elements but that their expression levels are stably maintained upon TET depletion due to the loss of TET-dependent repressive activities, such as SIN3A. Our findings implicate TET enzymes in the evolution of the TE-host relationship, contributing to exaptation processes and forming a hub for the so-called evolutionary arms race.

## Results

### TET1 binds to repetitive elements with diverse chromatin signatures

**Fig. 1:**
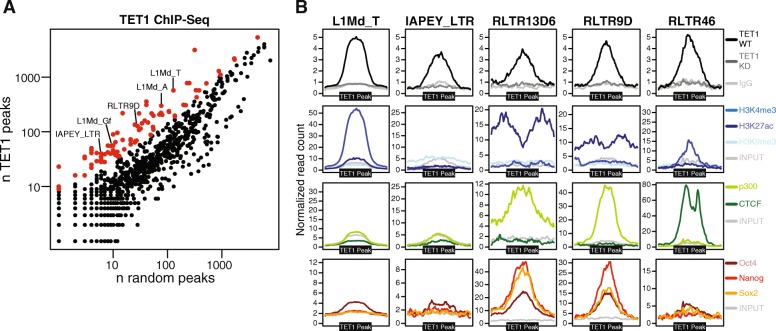
TET1 binds multiple TE classes. **a** Peaks from TET1 ChIP-seq data were overlayed with the RepeatMasker annotation to determine TE classes that are enriched in TET1 over a random control (highlighted in *red*). **b** Trend plots displaying the epigenomic profiles of TET1 peaks overlapping different TE classes

Previous studies have established that TET1 primarily binds at CpG-rich gene promoters in mouse ESCs [28, 30]. However, a thorough investigation of TET1 binding to repetitive elements was not conducted. To test whether TET1 binds to particular repeat classes, we uniquely mapped chromatin immunoprecipitation (ChIP) sequencing data [28] and calculated the proportion of TET1 peaks that overlapped each repeat class within the RepeatMasker annotation. When compared to a random control, several repeat classes were found to be significantly enriched for TET1 peaks, which included L1 elements, IAPEYs and a number of other LTR elements (Fig. 1a; Additional file 1). Similar results were obtained when we also included non-uniquely mapped reads, which can nonetheless be assigned a particular repeat class (Additional file 1). This ‘inclusive mapping’ strategy assigns reads with multiple hits of equal mapping quality to one of those locations at random and is useful to assess overall patterns of repeat classes with low mappability. We therefore used inclusive mapping on publicly available epigenomic data to characterise the identified TET1 targets. Interestingly, we found that TE-associated TET1 binding sites display markedly different chromatin compositions depending on TE class (Fig. 1b; Additional file 2: Figure S1A). TET1 peaks at L1 elements (L1Md_T, L1Md_A, L1Md_Gf) showed a pronounced enrichment for H3K4me3, a mark associated with active gene promoters, which is reminiscent of that seen in TET1-bound CpG-rich promoters. At IAPEY elements TET1 binding occurs in a region that is not enriched for H3K9me3 (Fig. 1b) and it is possible that more overt TET1 binding is impaired by the strong H3K9me3 enrichment seen elsewhere throughout these elements [33]. RLTR46 elements were enriched for CTCF, as previously reported [5], raising the possibility that TET1 activity at these TEs may help to maintain a hypomethylated state that is permissive for CTCF binding [34, 35]. Finally, we found that TET1 peaks overlapping a number of LTR elements (e.g. RLTR13D6, RLTR9D) were associated with marks of active enhancers (H3K27ac, p300), consistent with an enrichment of 5hmC at enhancer elements [34]. Moreover, these TE classes are bound by the pluripotency factors NANOG, OCT4 and SOX2 (collectively referred to as NOS from here onwards), as previously reported [5], suggesting that maintenance of an ESC-specific gene expression programme may be aided by TET1 binding to TE-derived enhancers. Analysis of ChIP-sequencing data for TET2 [36], which is also expressed in ESCs, showed that a subset of the TEs targeted by TET1 are seemingly also bound by TET2 (Additional file 2: Figure S1B). Altogether these data show that TET enzymes binds to multiple TEs in ESCs, with potentially different functional outcomes depending on the underlying epigenetics of each TE class.

### TET2 helps to maintain gene expression driven by TE-derived enhancers

**Fig. 2:**
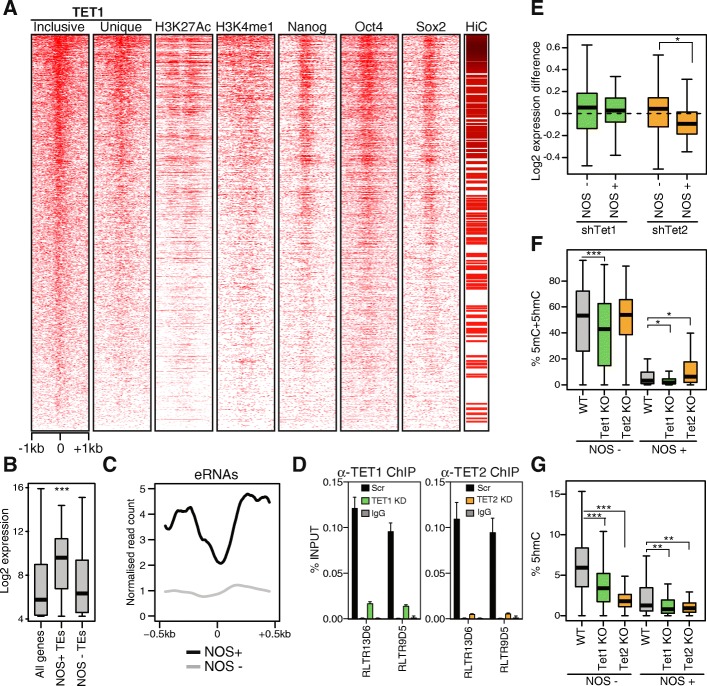
TETs target and control the activity of TE-derived ESC enhancers. **a** *Heatmap* of ChIP-seq and HiC data on individual copies of TE classes enriched for TET1 and NOS; data are uniquely mapped, except for TET1, where data from both inclusive and unique mapping are shown; each *line* in the HiC data depicts an interaction with a gene promoter. **b** RNA-seq data (average values from n = 5) shows that genes interacting with NOS+ TEs are expressed at a higher level than NOS- TEs of the same classes. **c** RNA profiles at enhancer-associated TE classes reveal bidirectional eRNAs emanating from TEs bound by NOS. **d** TET1 and TET2 ChIP-qPCR (representative replicate from n = 3), confirming their enrichment at TE classes associated with enhancer activity. **e** Changes in the RNA levels of genes interacting with TE-derived enhancers in TET1- or TET2-depleted ESCs; TET2 helps to maintain the expression of genes interacting with NOS+ TEs. **f** BS-seq data on WT, *Tet1* KO and *Tet2* KO cells shows that TET2 helps to maintain the hypomethylated state of NOS+ enhancer TEs. **g** TAB-seq data show that both TET1 and TET2 contribute to the 5hmC levels at enhancer-derived TEs. * *p* < 0.05, ** *p* < 0.01, *** *p* < 0.001, t-test (**b**, **e**) or Wilcoxon tests with Benjamini–Hochberg correction (**f**, **g**)

To investigate the putative role of TETs at TE-derived ESC enhancers, we first analysed uniquely mapped ChIP-seq data at individual copies of TE classes associated with TET1 and NOS. TET1 binding was strongly correlated with the levels of both enhancer-associated histone marks (H3K4me1, H3K27ac) and NOS (Fig. 2a). As enhancers regulate gene expression through direct contact with promoters, we also analysed three-dimensional chromatin conformation data from promoter capture Hi-C experiments [37]. We found that NOS-bound TE-derived enhancers were more likely to make contacts with gene promoters than their NOS-unbound counterparts (Fig. 2a). Importantly, genes interacting with NOS-bound TEs were expressed at higher levels than those interacting with NOS-unbound TEs of the same classes (Fig. 2b). RNA sequencing (RNA-seq) analysis on rRNA-depleted samples revealed short bidirectional enhancer RNAs (eRNAs) generated at NOS-bound TEs (Fig. 2c), adding further support to their role as active enhancers [38]. To test whether TET enzymes helped to maintain the activity of TE-derived enhancers, we depleted TET1 or TET2 in ESCs via lentiviral delivery of shRNAs. We first confirmed by ChIP that both TET1 and TET2 are enriched at TE classes associated with enhancer marks, with binding being lost upon depletion of TET1 or TET2 (Fig. 2d). We then performed RNA-seq to ask whether the expression of genes interacting with TE-derived enhancers was affected by TET depletion. Upon TET2 depletion there was a mild but significant downregulation of genes interacting with NOS-positive TEs (Fig. 2e). To test whether the effects of TET2 on enhancer activity were associated with changes in 5mC and/or 5hmC, we analysed BS-seq (which measures 5mC + 5hmC) and TAB-seq (which measures 5hmC) data from TET KO ESCs [39]. We found that NOS-positive TE copies had strikingly lower levels of 5mC than their NOS-negative counterparts, as expected from their enhancer activity (Fig. 2f). TET2 KO led to a more dramatic decrease of 5hmC at both groups of TEs than TET1 KO (Fig. 2g) and this was coupled with a specific increase in 5mC levels at NOS-positive TEs (Fig. 2f), which is in agreement with the gene expression data. These data show that TET enzymes help to maintain the expression of NOS-regulated genes by targeting specific retroelement classes and suggest that this occurs in a DNA methylation-dependent manner.

### TETs and 5hmC are enriched at the 5′ UTR of young L1 elements

**Fig. 3:**
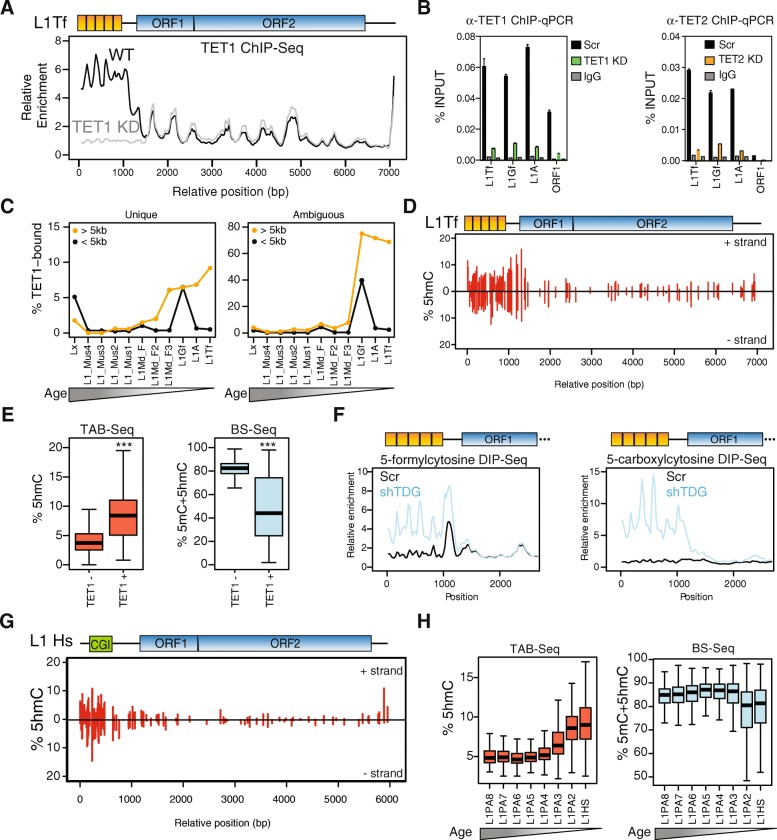
TETs bind to full-length, young L1s. **a** TET1 ChIP-seq data aligned to a full-length L1Tf element, revealing a specific enrichment at the 5′ UTR. **b** ChIP-qPCR confirms that TET1 and TET2 bind the 5′ UTR of the three active mouse L1 families, with reduced enrichment at the coding region (representative replicate from n = 3). **c** L1 clades were ordered according to evolutionary age and the proportion of overlapping TET1 peaks from either unique or inclusive mapping (the latter termed ‘ambiguous’ peaks) calculated; TET1 binding is restricted to full-length (>5 kb) young L1s. **d** TAB-seq data aligned to a L1Tf element reveal 5hmC enrichment at the 5’ UTR. **e** TAB-seq and BS-seq data analysis at L1Tf copies shows that TET1 binding is associated with higher 5hmC levels and concomitant lower 5mC (***p < 0.001, Wilcoxon test). **f** Profile of 5fC and 5caC enrichment along a full-length L1Tf; depletion of TDG leads to an accumulation of these modifications at the 5’ UTR. **g** Alignment of TAB-seq data from human ESCs to an active L1Hs element also shows an enrichment at the 5’ UTR. **h** Human L1 families were ordered according to evolutionary age and the levels of 5hmC/5mC extracted from TAB-seq and BS-seq data; young elements have higher 5hmC and lower 5mC levels

To analyse in more detail the binding of TET1 to L1 elements, we mapped the TET1 ChIP-seq data to a highly active L1Tf element (L1_Orl_ [40]), which revealed a striking enrichment of TET1 at the 5′ UTR of the element that was lost upon TET1 depletion (Fig. 3a). To validate and extend these findings, we first performed ChIP-qPCR on WT and TET-depleted ESCs, showing that both TET1 and TET2 bind to the 5′ UTR of L1A, L1Tf and L1Gf elements (Fig. 3b). We then investigated TET1 binding across different L1 subfamilies, ordering them by evolutionary age [16, 41]. Only full-length (>5 kb), evolutionarily young L1 elements had a substantial fraction of copies (5–10%) overlapping unique TET1 peaks (Fig. 3c). While many short L1Gf elements also appear to be bound by TET1, these are poorly annotated fragments that are often part of longer L1 copies. Given the difficulty in uniquely mapping ChIP-seq data to young L1s, we also calculated the overlap with TET1 peaks generated by inclusive mapping. We have termed these peaks ‘ambiguous’, as they cannot be confidently assigned to a specific copy when dealing with young L1s, but nonetheless reveal the maximum number of copies that may be TET1 bound. This strategy showed that nearly 80% of L1Gf, L1A and L1Tf elements have the potential to bind TET1 (Fig. 3c). Interestingly, the emergence and expansion of these young L1 families coincides with a loss of the repressive action of KAP1, which targets older L1s [16]. TET binding could constitute an additional mechanism by which young L1 elements have overcome the repressive mechanisms of the host. To test the involvement of TET binding in DNA methylation turnover at L1s, we analysed the levels of 5hmC throughout L1_Orl_, which revealed an enrichment at the 5′ UTR, matching the pattern of TET1 binding (Fig. 3d). Moreover, TET1-bound L1Tf copies display lower 5mC levels (and concomitantly higher 5hmC levels) than TET1-unbound copies (Fig. 3e), a pattern that is also found in other TET1-bound TE classes (Additional file 2: Figure S2). Analysis of 5fC and 5caC enrichment data [42] further revealed that, upon TDG depletion, these two marks substantially accumulate at the 5′ UTR of L1_Orl_ (Fig. 3f), showing that L1 elements undergo replication-independent DNA demethylation via 5mC oxidation.

We then asked whether a similar mechanism could be implicated in the regulation of human L1 elements. Using TAB-seq data from human ESCs [34], we uncovered a remarkably similar pattern of 5hmC at a highly active human L1 (L1.4 [43]), whereby the 5′ UTR displayed relatively high levels of this mark (Fig. 3g). Moreover, 5hmC enrichment across human L1 families mirrored our observations in the mouse, showing that only young, KAP1-unbound elements were enriched for 5hmC and this was accompanied by a decrease in 5mC levels (Fig. 3h). These observations show that, along independent evolutionary paths, L1 elements recruited TET enzymes and accumulated 5hmC at their 5′ UTR in a manner that correlates with their activity.

### TET-mediated L1 demethylation has no overall effect on expression

**Fig. 4:**
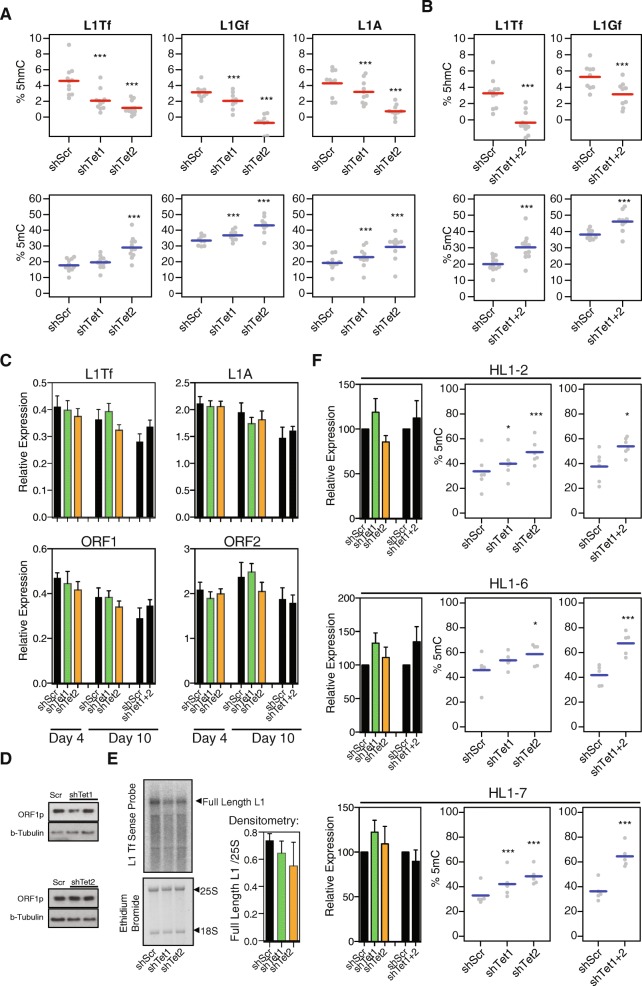
L1 expression is maintained upon TET-mediated demethylation. **a** Deep amplicon sequencing from oxBS-treated DNA was used to measure 5mC and 5hmC levels at young L1s in WT and TET-depleted ESCs; each data point represents the average value from three biological replicates at a given CpG within the amplicon. **b** Double TET1/TET2 knockdown does not lead to more pronounced effects on 5mC/5hmC than TET2 knockdown alone. **c** Quantitative reverse transcription polymerase chain reaction (RT-qPCR) data of TET1- and/or TET2-depleted ESCs, at four or ten days following lentiviral shRNA delivery (n = 6); no statistically significant differences are detected (t-test). **d** Western blot for ORF1p also shows no difference in expression at the protein level. **e** Representative northern blot for L1Tf, with averaged data from n = 4 quantified on the right; no statistically significant differences in the levels of full-length L1Tf are detected (t-test). **f** RT-qPCR (n = 6) and oxBS (n = 3) analysis of individual L1 copies reveals a similar pattern to that seen in the pool of all L1s, with no expression differences despite increased 5mC levels. * *p* < 0.05, *** *p* < 0.001, ANOVA with post-hoc Tukey test

The above data suggest that TET enzymes help to maintain L1 elements relatively hypomethylated in ESCs. We therefore measured 5mC and 5hmC levels at the 5′ UTR of L1s in TET-depleted ESCs using deep sequencing of amplicons from oxidative bisulfite (oxBS)-treated DNA [32, 44]. TET1 depletion led to a decrease of 5hmC at young L1 elements (L1Tf, L1A, L1Gf), which was accompanied by a significant increase in 5mC levels at L1A and L1Gf elements (Fig. 4a). TET2 depletion had a more dramatic effect for all three L1 classes, leading to a complete loss of 5hmC and an increase of 5mC levels of around 10% (Fig. 4a). To test for redundancy between the two TET enzymes, we performed double knockdown (DKD) of TET1 and TET2 and measured 5mC/5hmC levels. Compared to TET2 depletion alone, DKD did not lead to an additional increase in 5mC levels (Fig. 4b). We also analysed TAB-seq data from WT, *Tet1* KO and *Tet2* KO ESCs [39], which showed that the changes in 5mC/5hmC levels are largely restricted to the 5′ UTR region of L1Tf elements, and corroborated the increase in 5mC levels in both TET1- and TET2-depleted cells (Additional file 2: Figure S3A and B). Finally, we re-analysed BS-seq data from WT and *Tet1*/*Tet3* double knockout blastocysts, confirming that TETs maintain L1 hypomethylation in vivo (Additional file 2: Figure S3C) [45]. These data show that TET1 and TET2 are major regulators of DNA modifications at young L1s, helping to maintain low 5mC.

Given that loss of DNA methylation in DNMT KO ESCs leads to L1 derepression [15, 19], we predicted that TET depletion would cause a downregulation of L1s. Surprisingly, however, quantitative reverse transcription polymerase chain reaction (RT-qPCR) analyses showed that the expression levels of L1 elements were unaffected in TET1-depleted or TET2-depleted ESCs, both at four and ten days after lentiviral infection (Fig. 4c). L1 expression was also stable upon DKD of both TETs (Fig. 4c). Analysis of our RNA-seq data supported these observations and further revealed that no TET1-bound TE classes were deregulated in TET-depleted ESCs (Additional file 2: Figure S4A and B); in contrast, substantial changes in the expression of single-copy genes were readily detectable (Additional file 2: Figure S4C). RNA-seq data from WT and *Tet1*/*Tet3* double knockout blastocysts further supported these findings (Additional file 2: Figure S4D) [45]. In line with the RNA data, we detected no alterations in the levels of ORF1p protein (Fig. 4d). Additionally, using northern blot we found no significant differences in the levels of full-length L1Tf elements (Fig. 4e). We considered the possibility that changes in the expression of individual L1 copies that gained 5mC may be undetectable when analysing the whole L1 RNA pool. To test this hypothesis, we designed specific primers for RT-qPCR and oxBS that target three individual full-length L1 loci in the mouse reference genome (referred to here as HL1-2, HL1-6 and HL1-7; see ‘Methods’ for details). All tested L1 copies showed a significant increase in 5mC levels upon DKD of both TETs or single knockdown of TET2, with two of them also displaying changes upon TET1 depletion (Fig. 4f). Despite these differences, none of these L1 copies had significantly altered expression levels upon depletion of TET1, TET2 or both enzymes (Fig. 4f).

Overall, these results demonstrate that TET-mediated changes in DNA methylation at L1s do not translate into expression differences, indicating that although DNA methylation is an important regulator of L1 repression in ESCs, other layers of regulation can play equally prominent roles.

### TET-dependent epigenetic activities keep L1s repressed

**Fig. 5:**
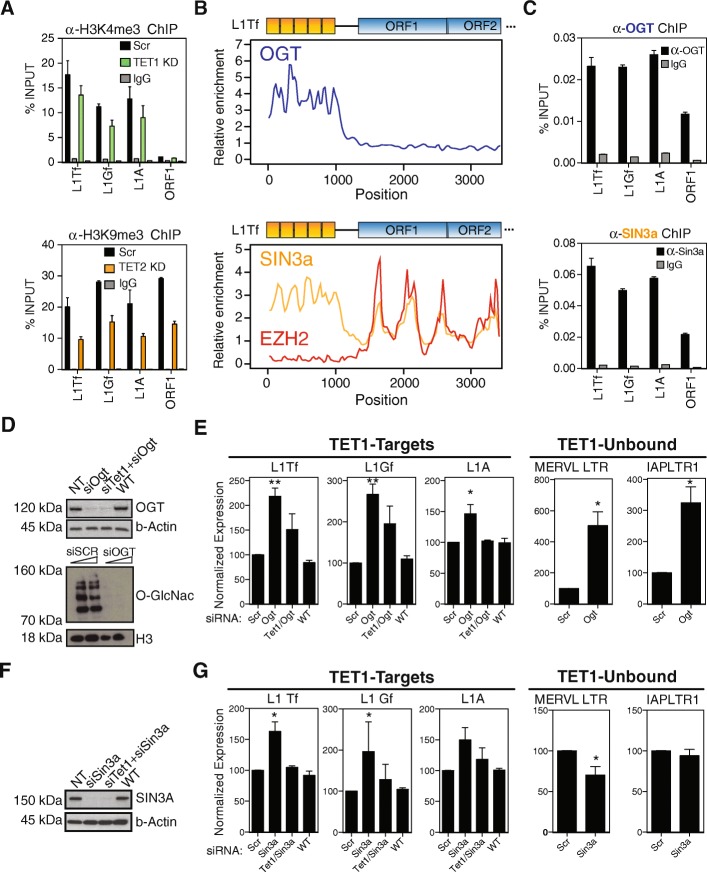
TET-dependent epigenetic activities repress L1 expression. **a** TET1 depletion leads to a relatively small loss of H3K4me3, whereas TET2-depleted cells have a pronounced loss of H3K9me3 (representative replicate from n = 3–7; see Figure S6B). **b** ChIP-seq data reveal that OGT and SIN3A, but not EZH2, are enriched at the 5′ UTR of L1Tf elements. **c** ChIP-qPCR analysis for OGT and SIN3A confirms the binding of both proteins at the 5′ UTR of young L1s (representative replicate from n = 4). **d** OGT was knocked down in ESCs by siRNA, leading to efficient depletion of the protein, as well as complete loss of cellular O-GlcNAc. **e** OGT depletion leads to L1 derepression at the RNA level, with partial effects also visible in TET1/OGT double knockdown cells; TE classes that are not TET1 targets are also upregulated (n = 8 for Ogt KD and n = 3 for Tet1/Ogt KD). **f** SIN3A was knocked down in ESCs by siRNA, with efficient loss of protein expression. **g** SIN3A depletion leads to L1 derepression at the RNA level, and this depends on TET1 expression; the effect is restricted to TET1-bound TE classes (n = 6 for Sin3a KD and n = 4 for Tet1/Sin3a KD). * *p* < 0.05, ** *p* < 0.01, paired t-test comparing to scrambled control

The above data raised the possibility that additional mechanisms compensate for changes in DNA methylation in TET-depleted cells to ensure L1 repression. Given the known involvement of small RNAs in human L1 regulation [18, 46], we first asked whether small RNA pathways could underlie the stability of L1 expression levels in TET-depleted cells. Small RNA-seq of WT and TET-depleted cells revealed no differences in the amount of L1-derived small RNAs (Additional file 2: Figure S5), suggesting that these pathways are not directly involved in the maintenance of L1 RNA levels upon TET depletion. We next asked whether TET enzymes regulate other aspects of L1 chromatin. We used ChIP-seq data from *Tet1* and *Tet2* KO ESCs and assessed the levels of several histone modifications at L1-associated TET1 binding sites (Additional file 2: Figure S6A). The most notable change was a loss of H3K9me3 in *Tet2* KO ESCs, which we validated by ChIP-qPCR in our knockdown cells (Fig. 5a; Additional file 2: Figure S6B). Loss of H3K9me3 at L1s, which is deposited by SUV39H, leads to transcriptional derepression [47], potentially counteracting the increase in 5mC in TET2-depleted cells. In *Tet1* KO cells, however, no pronounced alterations in the levels of the tested histone modifications were seen in ChIP-seq data (Additional file 2: Figure S6A). Indeed, by ChIP-qPCR we could only detect a relatively minor loss of H3K4me3 at L1 elements upon TET1 depletion (Fig. 5a; Additional file 2: Figure S6B), as well as impaired OCT4 binding (Additional file 2: Figure S6C). Both of these changes would be expected to further contribute to L1 downregulation upon TET1 depletion, together with increased 5mC.

We argued that any compensatory repressive mechanism that depends on TET1 is likely to be mediated by known interacting partners, namely OGT, SIN3A and the PRC2 complex [27–29]. Publicly available ChIP-seq data revealed that both OGT and SIN3A are enriched at the 5′ UTR of L1 elements, while members of the PRC2 complex are undetectable (Fig. 5b). We confirmed the enrichment of OGT and SIN3A at the 5′ UTR of young L1s by ChIP-qPCR (Fig. 5c). To test the effect of OGT on L1 expression, we depleted ESCs of OGT by siRNA, which led to a global loss of O-GlcNAc (Fig. 5d). Interestingly, OGT depletion caused an upregulation of L1 elements and this effect was partly dependent on TET1 expression (Fig. 5e). We confirmed that L1 derepression involved full-length elements and that ORF1p protein levels were also raised (Additional file 2: Figure S7A and B). However, we noted that TEs that are not bound by TET1 were also derepressed, suggesting that the effect of OGT depletion on L1s may be indirect (Fig. 5e). We then raised cellular levels of O-GlcNAc by inhibiting O-GlcNAc hydrolase (OGA), which led to a small increase in the expression of L1s, but not of TET1-unbound TEs (Additional file 2: Figure S7C and D). The seemingly conflicting outcomes of OGT depletion and OGA inhibition likely reflect the broad and complex roles that O-GlcNAc plays in chromatin regulation, with both activating and repressive outcomes [48, 49]. Nonetheless, it remains possible that cycling of O-GlcNAc through OGT and OGA plays a direct role in L1 silencing. To test whether recruitment of SIN3A to L1s is involved in their regulation, we used siRNA/shRNA-mediated depletion of SIN3A (Fig. 5f). This caused a 50–70% increase on the expression levels of young L1s, whereas TEs unbound by TET1 remained unaffected (Fig. 5g). Moreover, the effect on L1s was TET1-dependent, as the upregulation observed on SIN3A KD samples was lost when we knocked down both SIN3A and TET1, which is in line with the TET1-dependent recruitment of SIN3A to chromatin [28]. We also confirmed that SIN3A-mediated L1 derepression involved full-length elements and a concomitant increase of ORF1p protein levels (Additional file 2: Figure S7A and B). This result is, to the best of our knowledge, the first time that SIN3A has been implicated in TE regulation. Interestingly, SIN3A binding is also seen at the 5′ UTR of human L1s in ESCs (Additional file 2: Figure S8), raising the possibility that human L1s are also kept repressed by TET-dependent deposition of SIN3A. Our data show that there are TET-dependent repressive activities that likely counteract oxidative DNA demethylation at L1 elements. Indeed, we show that, in a SIN3A-null context, TET1 is a positive regulator of L1 expression. Our work uncovers TET enzymes as epigenetic hubs with dual roles in L1 regulation.

## Discussion

**Fig. 6:**
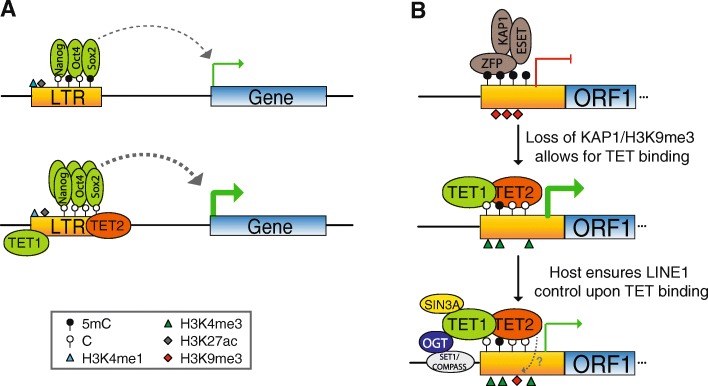
Models of TET-dependent regulation of TEs. **a** TE-derived ESC enhancers interact with highly expressed genes; TET binding is associated with lower 5mC levels, increased NOS binding and higher gene expression, which could have played a role in the exaptation of these TEs by the host genome. **b** L1 elements bound by KRAB-ZFP/KAP1 do not bind TETs and have high 5mC levels; loss of KAP1 binding (in currently active L1s or at different points during L1 evolution) would allow for TET binding, which would drive DNA demethylation and promote LINE-1 expression and expansion; upon TET binding, recruitment of other TET-dependent epigenetic modifiers (e.g. SIN3A) ensure that L1 expression is kept under control, constituting host defence strategies that could partly have evolved from the need to maintain genome stability

We have shown that TET enzymes bind multiple TEs, driving epigenetic alterations that impact on TE and gene expression. Importantly, TET binding displays a TE-specificity that implicates these enzymes in the evolution of the host-TE relationship. Our detailed bioinformatic characterisation revealed that there is no single epigenetic signature underlying TET1 binding to TEs and instead TET1-bound TEs display a myriad of epigenetic profiles that suggest distinct functions. Namely, TETs bind exapted TEs that are used by the host as CTCF sites (RLTR46) and enhancer elements (RLTR13D6 and others), potentially involving TETs in the exaptation process. Indeed, our data show that TET2 helps to control the expression of genes interacting with TE-derived enhancers, likely by maintaining a hypomethylated state that is permissive for TF binding. TET2 binding to specific TEs seems to have conferred them with an additional means for affecting gene expression that could have contributed to their exaptation by the host genome (Fig. 6a).

At L1 elements, TET binding displays a striking pattern whereby it targets exclusively the 5′ UTR of young, active L1 clades. Moreover, the same pattern is apparent in human ESCs (as judged by the presence of 5hmC), which is suggestive of an evolutionarily convergent mechanism that allows for L1 activation and expansion. Parallel evolution of L1 silencing mechanisms is also seen for KAP1 action, which targets and silences older L1 elements in both mouse and human through the binding of KRAB-ZFPs [16, 17]. Interestingly, none of the young TET1/5hmC-enriched L1s are KAP1 targets. KAP1 binding leads to recruitment of SETDB1 and deposition of H3K9me3, which has been suggested to impair TET1 binding at TEs [33]. Therefore, the evolutionary escape of L1s from KAP1-mediated repression may have created permissive conditions for TET binding, which in turn led to DNA demethylation (Fig. 6b). Accordingly, we found that TET depletion leads to increased methylation at the 5′ UTR of young L1s. It is possible that the scale of this effect is dependent on cell culture conditions, as these are known to deeply affect DNA methylation levels in ESCs [21]. We note that L1 methylation levels in ESCs grown with standard serum-containing medium, as used for all our experiments, closely match those seen in blastocysts and inner cell mass cells (Additional file 2: Figure S9). In contrast, cells grown with the so-called ‘2i’ formulation (with or without vitamin C) display substantially lower L1 methylation levels (Additional file 2: Figure S9), suggesting that the epigenetic modifiers targeting L1s in 2i cells differ to those acting in vivo.

Given that young L1s are derepressed in DNMT-null ESCs [15, 16], TET-mediated DNA demethylation is expected to activate L1 expression. However, our data clearly show that TET depletion has no overall effect on L1 expression levels, potentially arguing against a role of TET recruitment in TE expansion. Mechanistically, the maintenance of L1 expression upon TET-mediated demethylation suggests that DNA methylation may not play as prominent a role in the regulation of these TEs as previously thought. It is possible that the L1 derepression seen in DNMT mutants [15, 16, 19] are independent of the enzymes’ catalytic activity [50], although ESCs expressing catalytically inactive DNMT1 fail to repress IAPs [51]. Alternatively, TETs may drive other catalytically independent alterations to chromatin that compensate for changes in DNA methylation. In line with this hypothesis, we found that TET2 depletion leads to a reduction of H3K9me3 levels at L1s and that TET1 recruits the SIN3A co-repressor complex, which in turn plays a role in L1 silencing. TET1 also recruits OGT to L1s and our data indicate a possible role for O-GlcNAc turnover in L1 repression. Interestingly, SIN3A is modified by OGT to synergistically repress transcription [52], raising the possibility that the OGT-mediated and SIN3A-mediated repressive effects on L1s are mechanistically linked. However, OGT may also affect TE expression through RNA Pol II [53, 54] or other effects on chromatin [48, 49].

Could TET-dependent repressive activities constitute a host strategy for ensuring L1 silencing upon TET-mediated DNA demethylation? In one possible evolutionary scenario, as a newly formed L1 clade (not necessarily the most recent ones) escaped from the repressive action of KRAB-ZFP/KAP1, permissive TET-binding conditions would have been created that would lead to DNA demethylation, contributing to L1 expansion (Fig. 6b). Selective pressure to maintain genome stability could then have driven the evolution of TET-dependent host strategies to control L1 expansion, such as sequestration of epigenetic silencing activities (e.g. SIN3A) by TET enzymes (Fig. 6b). In this scenario, TETs would have served as ‘double agents’ in an arms race between L1s and the host genome. As each subsequent emerging L1 family escaped KRAB-ZFP/KAP1 repression, the activating effects of TET binding would have been dampened by the accompanying repressive activities. More effective and permanent silencing of an active L1 family would then require the recognition by a novel KRAB-ZFP [17]. In an alternative scenario, the main selective pressures driving the interactions between TETs and epigenetic silencers could have come from elsewhere. Yet the added need to maintain control of L1 expression during preimplantation could have arguably re-enforced these epigenetic relationships and further contributed to their evolution and fine-tuning.

It remains possible that in other cell types TET-mediated control of DNA methylation regulates L1 transcription due to differences in the underlying epigenome. For example, TET1 and TET2 silencing during early ESC differentiation may be important to ensure full L1 repression through DNA methylation. And in neurons, where 5hmC levels are particularly high [55], TET-mediated DNA demethylation could help drive the high levels of L1 expression seen therein [56, 57].

## Conclusions

We have unveiled several novel roles of TET enzymes in the regulation of TEs, both catalytically dependent and independent. TETs mediate DNA demethylation of several TEs, but also serve as a hub for additional epigenetic activities, both of which impact on the evolution of the TE-host relationship. The intriguing dual nature of TET action on L1 elements appears to reflect the competing interests of host and TEs, raising new questions on TE-driven evolution of epigenetic mechanisms.

## Methods

### ESC culture, gene knockdown and PUGNAc treatment

E14 ESCs were grown in feeder-free conditions using DMEM-based medium with 15% FBS and 1000 U/mL ESGRO LIF (Millipore). For shRNA-mediated gene knockdown, ESCs were infected with viral particles carrying pLKO.1 constructs harbouring gene-specific shRNAs or a non-targeting sequence (Scr: CCTAAGGTTAAGTCGCCCTCGCTC; shTET1: TTTCAACTCCGACGTAAATAT, TRCN0000341848; shTET2: TTCGGAGGAGAAGGGTCATAA, TRCN0000250894). After 24 h, cells were selected with 10 μg/mL puromycin or 50 μg/mL hygromycin for 3–9 days (see ‘Results’). For siRNA-mediated knockdown, cells were transfected twice (second transfection two days after the first one) with gene-specific (see below) or non-targeting siRNAs (Dharmacon siGENOME Non-Targeting siRNA #2 Cat.D-001210-02-20) using Lipofectamine 3000 (Thermo Scientific, Cat. L3000008) and collected four days post transfection. *Tet1* (Dharmacon siGENOME Mouse *Tet1* Cat. D-062861-01-0020) and *Sin3a* siRNAs (Dharmacon Custom siRNA ON-TARGET Cat. CTM-220747, sequence: gctgttccgattgtccttaaa) were used at 125 nM, whereas *Ogt* esiRNAs (Sigma-Aldrich, Cat. EMU006701) were used at 50 nM. Knockdown was confirmed by RT-qPCR and western blot. PUGNAc (Sigma-Aldrich, Cat. A7229) treatment was performed either with 100 μM of DMSO-dissolved PUGNAc or matched amount of DMSO in control conditions and its efficiency was tested by western blot. A list of antibodies used can be found in Additional file 3.

### RNA isolation, RT-qPCR and northern blot

RNA was extracted using Quick-RNA™ MiniPrep (Zymo Research D7003) and DNAse treated in-column either with the DNAse provided on the kit or with the TURBO DNA-free™ Kit (Ambion, AM1907). RNA (1 μg) was retrotranscribed using SuperScript® III Reverse Transcriptase (Invitrogen, Cat. 18080044) and the cDNA was diluted 1/10 for qPCRs using MESA BLUE MasterMix (Eurogenentec, 10-SY2X-03 + NRWOUB) on a LightCycler® 480 Instrument II (Roche). A list of primers used can be found in Additional file 3: Table S2. For northern blotting, 0.5 μg of glyoxylated total RNA was separated on a 1.2% BPTE gel, then blotted and probed as described in [58]. RNA probes were amplified from mouse genomic DNA using the primers detailed in Additional file 3.

### Chromatin immunoprecipitation

ChIP was performed as described in [59], with modifications. For the detection of TFs (TET1, TET2, SIN3A, OGT, OCT4), cells were fixed with an initial cross-linking step of 45 min with 2 mM Di(N-succinimidyl) glutarate (Sigma-Aldrich Cat. 80424) in PBS at room temperature, followed by a PBS wash and a second fixation step of 12 min with 1% formaldehyde in DMEM. For histone ChIPs (H3K4me3, H3K9me3, H3) a single cross-linking step with 1% formaldehyde for 15 min was used. After quenching with glycine, washes and lysis, chromatin was sonicated using a Bioruptor Pico from Diagenode, on a 30 s on/off cycle for 12 (TFs) or eight (histones) cycles. Immunoprecipitation was performed using 150 μg of chromatin and 7.5 μg of antibody (TFs) or 30 μg of chromatin and 5 μg of antibody (histones). Final DNA purification was performed using the GeneJET PCR Purification Kit (Thermo Scientific. Cat. K0701) and elution in 80 μL of elution buffer. This was diluted 1/10 and analysed by qPCR, using the KAPA SYBR® FAST Roche LightCycler® 480 2X qPCR Master Mix (Kapa Biosistems, Cat. KK4611). A list of antibodies and primers used can be found in Additional file 3.

### RNA-seq and small RNA-seq

Ribosomal RNA-depleted RNA-seq libraries were prepared from 400–600 ng of total RNA using the low input ScriptSeq Complete Gold Kit (Epicentre). For small RNA-seq, RNA was isolated with QIAzol (QIAGEN) and libraries prepared from 400–600 ng of RNA using the NEBNext small RNA library prep kit (NEB), followed by size selection (~120–150 bp, including adaptors) using gel extraction. Libraries were sequenced on an Illumina NextSeq 500 with single-end 75 bp reads.

### Oxidative bisulphite sequencing

Deep sequencing of PCR products from BS-converted and oxBS-converted DNA was performed as previously described [44]. Briefly, precipitated DNA (without glycogen) was resuspended in water and further purified using Micro Bio-Spin columns (Bio-Rad), after which half of the DNA was oxidised with 15 mM KRuO_4_ (Alpha Aesar) in 0.5 M NaOH for 1 h. Following bisulphite conversion of both DNA fractions with the EpiTect Bisulfite kit (QIAGEN), a two-step PCR amplification was used: a first PCR amplifies the region of interest and adds part of the sequencing adaptors; a second PCR on pooled amplicons then completes the adaptors and adds sample barcodes, allowing for multiplexing (see primers in Additional file 3). Paired-end sequencing of pooled samples was done using an Illumina MiSeq.

### Individual L1 elements

For RT-qPCR and oxBS analysis of individual L1s in the mouse reference genome, assays were first designed for several full-length elements with uniquely mapped reads and high 5hmC levels in TAB-seq data. We then selected elements whose RT and oxBS amplicons were validated by Sanger sequencing and for which the abundance in genomic DNA was close to that of single copy genes, as judged by qPCR. Elements have the following mm9 coordinates: chr2:125230942–125237372 (HL1-2; contains L1Tf monomers), chr5:70814798–70821450 (HL1-6; L1Tf monomers), chr6:107895070–107901677 (HL1-7; L1A monomers).

### High-throughput sequencing data processing

External datasets were downloaded from GEO (Additional file 4). Reads were trimmed using Trim_galore! v0.3.3 with default parameters, except for small RNA-seq, where the options ‘-q 0 --length 15’ were used. Alignments were performed either to the whole mouse genome (mm9), to the L1_Orl_ sequence (accession number D84391) or the L1.4 sequence (accession number L19092). ChIP-seq and small RNA-seq data were aligned using Bowtie2 v2.1.0 [60] with default parameters, which assigns reads with multiple hits of equal mapping quality to one of those locations at random (inclusive mapping). To obtain uniquely mapped data, the output SAM file was filtered using a custom script. ChIP-seq peak detection was performed using the MACS algorithm implemented within Seqmonk. RNA-seq data were aligned using Tophat v2.0.9 [61] with -g 1 option, which yields inclusive mapping. BS-seq and TAB-seq data were aligned to representative L1 elements (as above) using Bismark [62]; for genome-wide analysis, processed CpG calls from uniquely mapped data were obtained directly from GEO (see Additional file 4). OxBS data were aligned with Bismark to a custom genome containing the amplicon sequences; only CpGs covered by at least 100 reads were used to calculate 5mC/5hmC levels.

### TET1 ChIP-seq analysis

To detect TE classes that were enriched for TET1 binding events, the number of TET1 peaks (from unique or inclusive mapping) overlapping each TE class (using the Repeatmasker annotation) were summed. This was compared with the overlaps obtained using a matched list of regions (equal number and lengths than the TET1 peaks) randomly distributed anywhere in the genome (for inclusive mapping) or within mappable regions of the genome, as defined by the uniqueome [63] (for unique mapping).

### RNA-seq analysis

Raw read counts for each gene or repeat (from RepeatMasker annotation) were generated in Seqmonk with the RNA-seq quantitation pipeline. Reads from repeats belonging to the same class were pooled. DESeq2 was used for differential expression analysis and for generating normalised gene and repeat expression values. To couple TE-derived enhancers to gene expression values, promoter capture Hi-C data from ESCs [37], as processed by CHiCAGO [64] (kindly provided by Paula Freire-Pritchett, Babraham Institute), was used.

## Additional files

Additional file 1: List of TET1-bound TEs using either unique or inclusive mapping. (XLSX 51 kb)
Additional file 2:
**Figures S1 to S9.** (PDF 1217 kb)
Additional file 3: List of antibodies and primers. (XLSX 34 kb)
Additional file 4: Accession numbers of external datasets. (XLSX 41 kb)
