# Supplementary material for: TET-dependent regulation of retrotransposable elements in mouse embryonic stem cells
Source: Genome Biol. 2016 Nov 18;17:234. doi: 10.1186/s13059-016-1096-8 (PMC5116139; doi:10.1186/s13059-016-1096-8)
Supplement: Additional file 2: — Figures S1 to S9. (PDF 1217 kb) [file 13059_2016_1096_MOESM2_ESM.pdf]

Figure S1

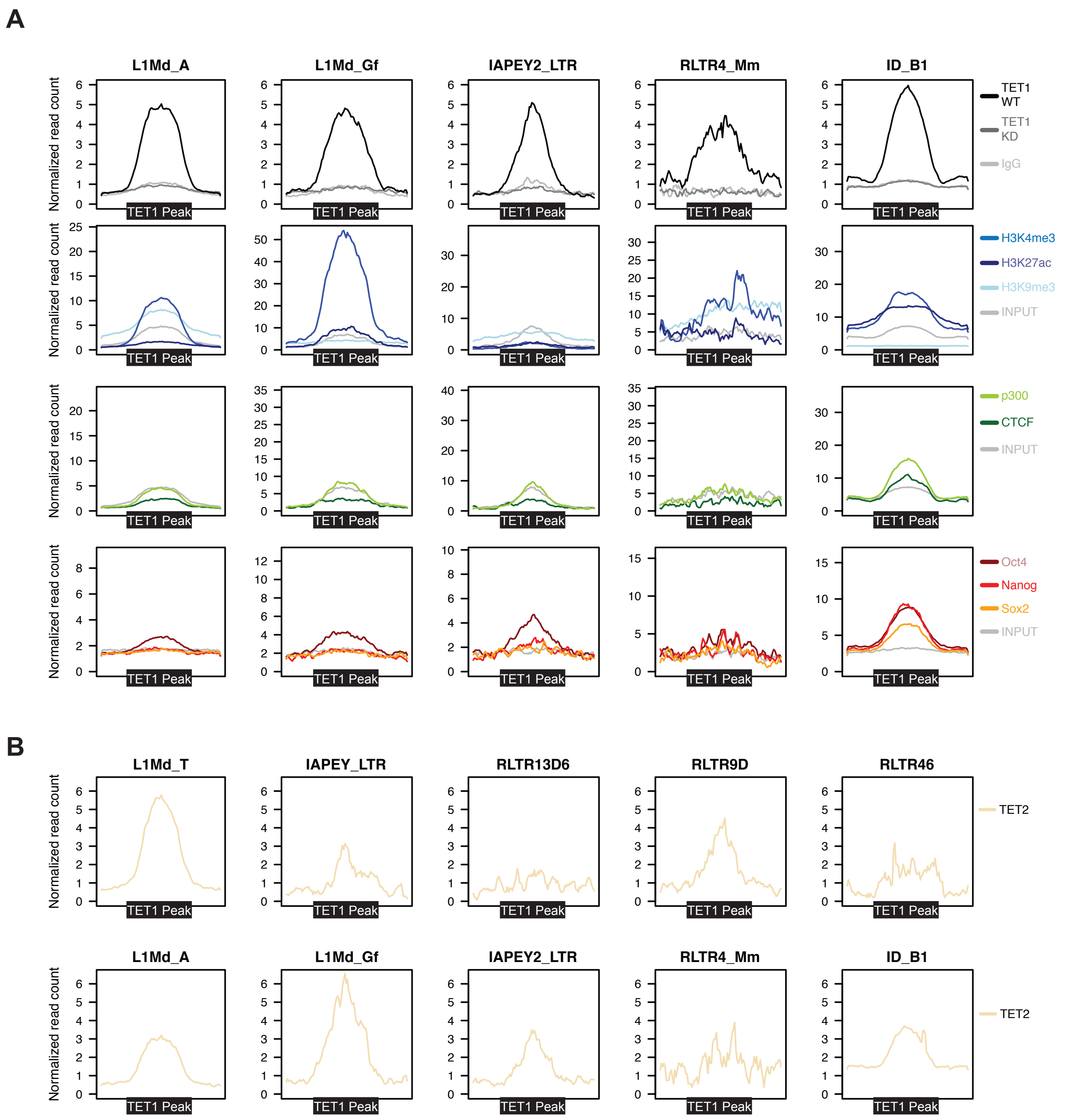

Figure S1 – Additional trend plots at TET1 peaks overlapping TEs. **A)** ChIP-seq data displaying the epigenomic profiles of TET1 peaks overlapping different TE classes. **B)** Trend plots from TET2 ChIP-seq data at TE-overlapping TET1 peaks.

Figure S2

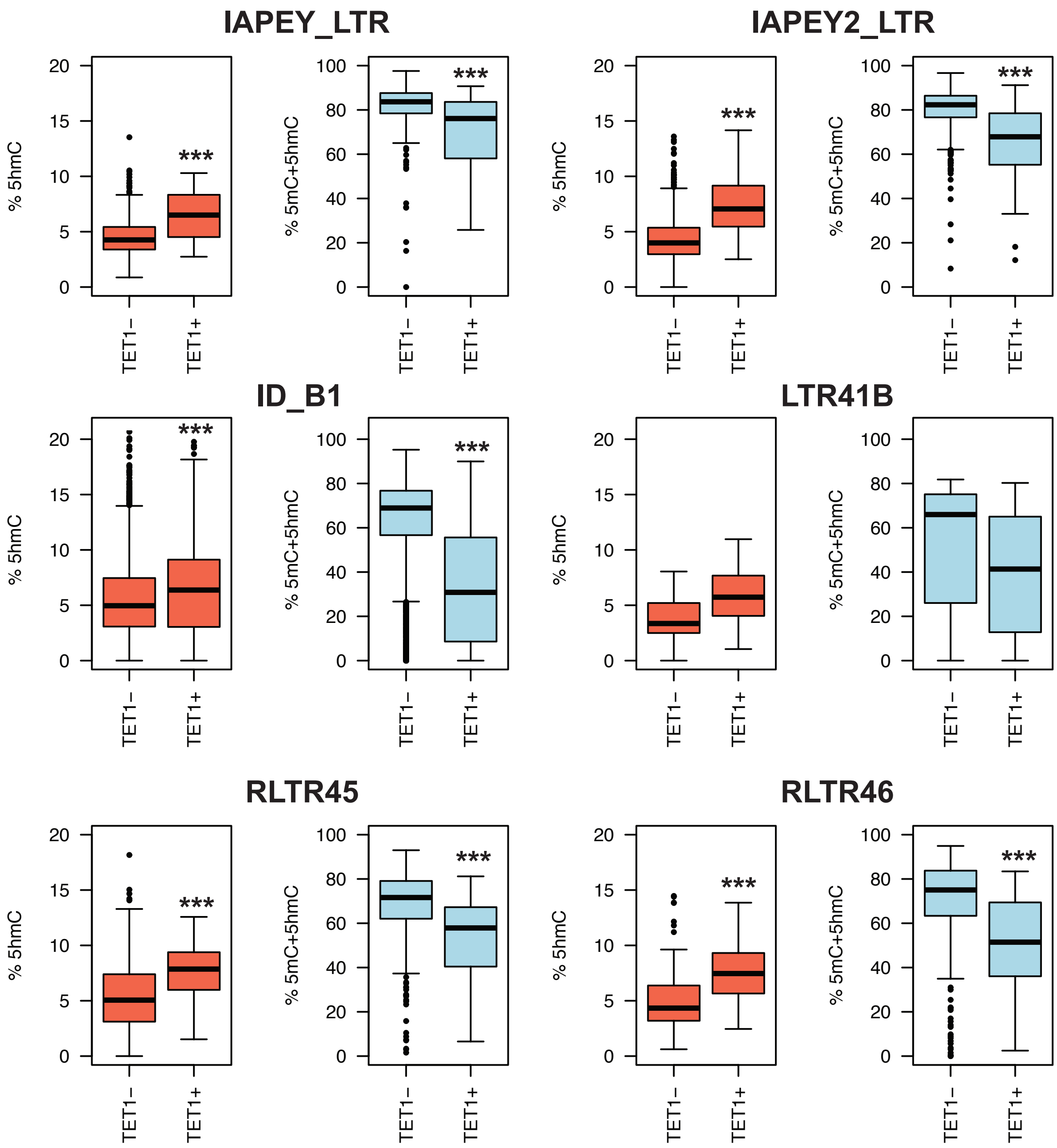

Figure S2 – Analysis of BS-seq and TAB-seq data at additional TE classes. TET1-bound copies tend to have higher 5hmC levels and concomitantly lower 5mC. *** p<0.001, Wilcoxon test.

Figure S3

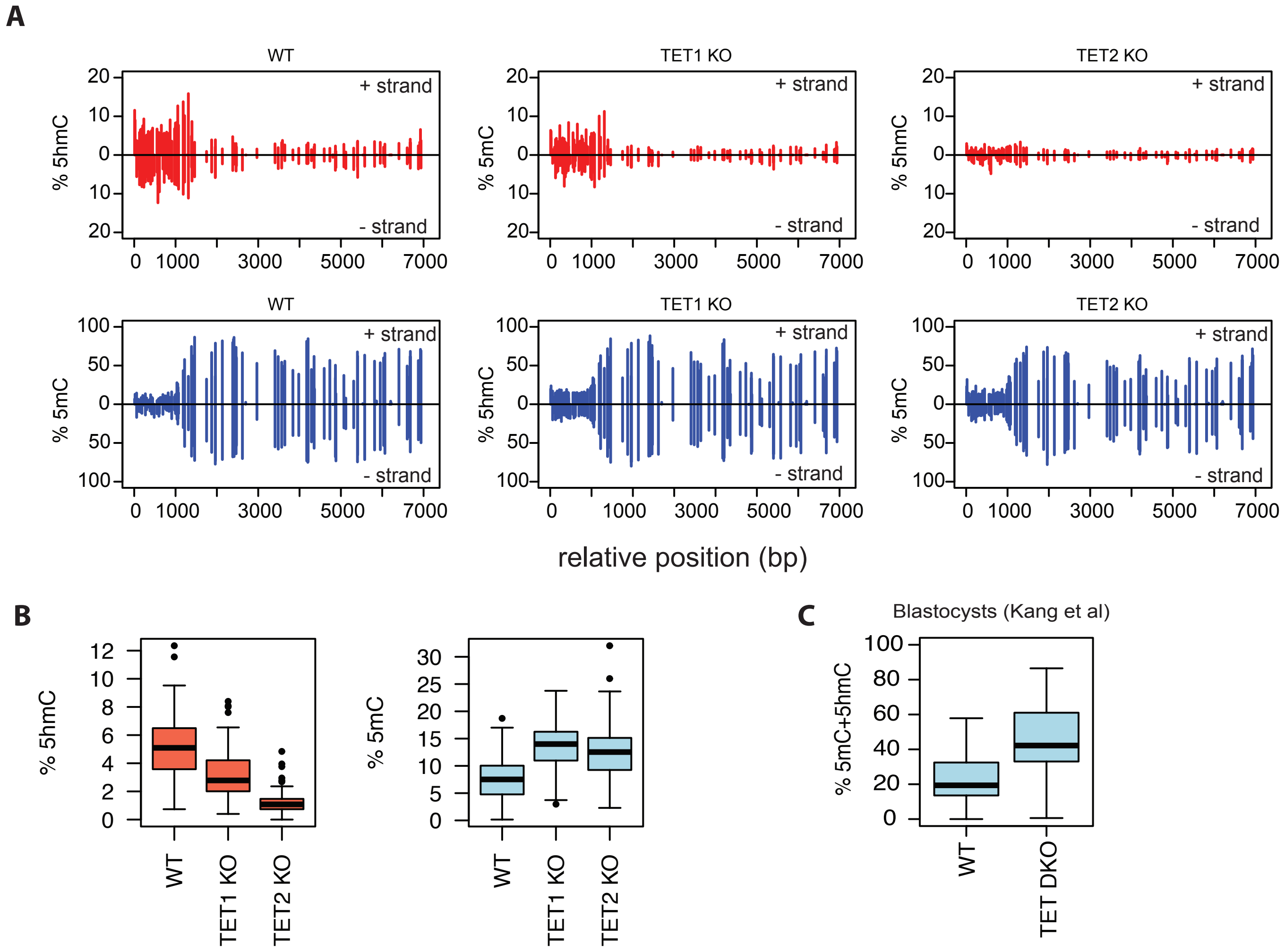

Figure S3 – BS-seq and TAB-seq data at L1Tf elements. **A)** Data from WT, *Tet1* KO and *Tet2* KO ESCs were aligned to a L1Tf element, confirming that TET enzymes maintain the 5' UTR of L1Tf elements hypomethylated, with TET2 being the main contributor to 5hmC levels. **B)** 5mC/5hmC levels within the 5' UTR were extracted from the L1Tf profile in (A). **C)** A similar analysis of the 5' UTR of L1Tf was done using BS-seq data from WT and *Tet1/Tet3* double knockout blastocysts.

Figure S4

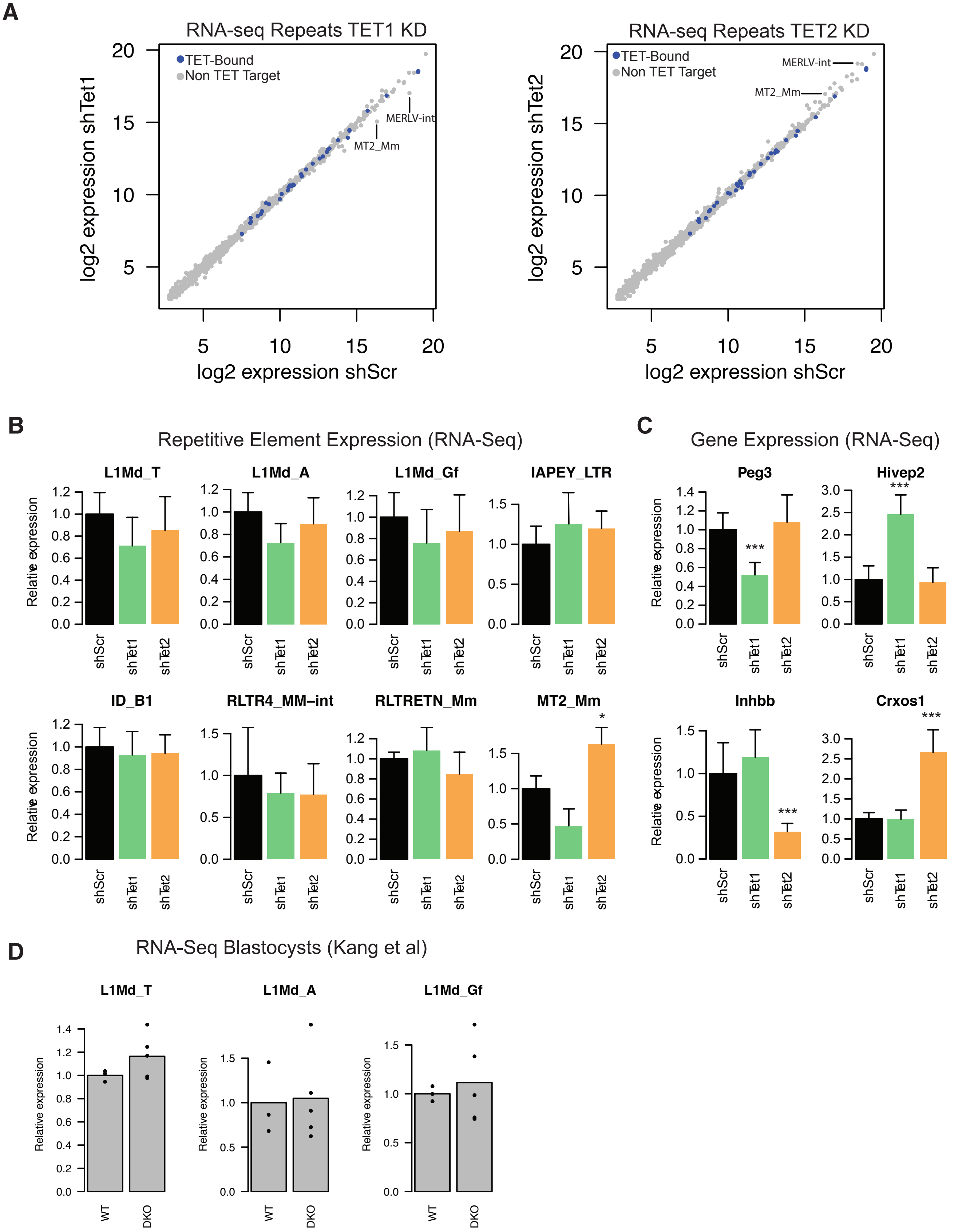

Figure S4 – RNA-seq data analysis. **A)** The total relative amount of RNA from each repeat class was plotted for control and TET-depleted ESCs; TET1-bound TE classes are highlighted in blue; only the LTRs of MERVL elements (MT2), which are not TET1 targets, were found to be differentially expressed. **B)** Average expression levels for selected TE classes were extracted from RNA-seq data from five biological replicates. **C)** Examples of genes found to be differentially expressed in TET1- or TET2-depleted ESCs. RNA-seq was performed in 5 biological replicates. **D)** L1 expression levels extracted from RNA-seq data from WT and *Tet1/Tet3* double knockout blastocysts. * $p<0.05$, *** $p<0.001$, corrected p-values from DESeq2.

Figure S5

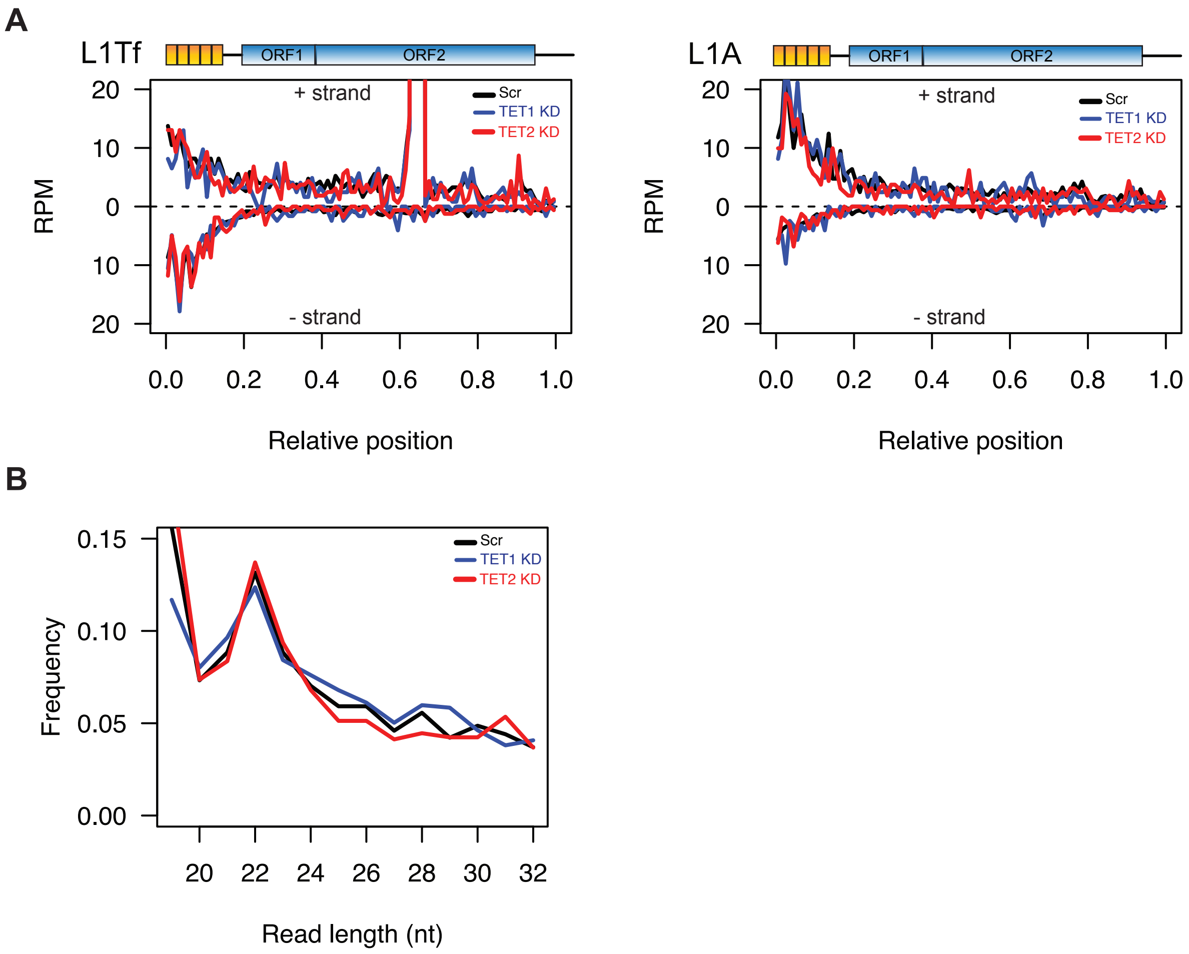

Figure S5 – Small RNA-seq analysis. **A)** Reads from RNAs ranging 19-32 nt in size were aligned using inclusive mapping and the total levels of small RNAs overlapping L1 elements plotted (note that the peak in the middle of L1Tf is a mapping artefact); no changes were observed upon TET depletion. **B)** Reads mapping to the 5’ UTR of young L1s (L1A, L1Tf, L1Gf) were analysed with respect to their size distribution; no changes are seen in these profiles in TET-depleted cells. Small RNA-seq was performed in 2 biological replicates.

Figure S6

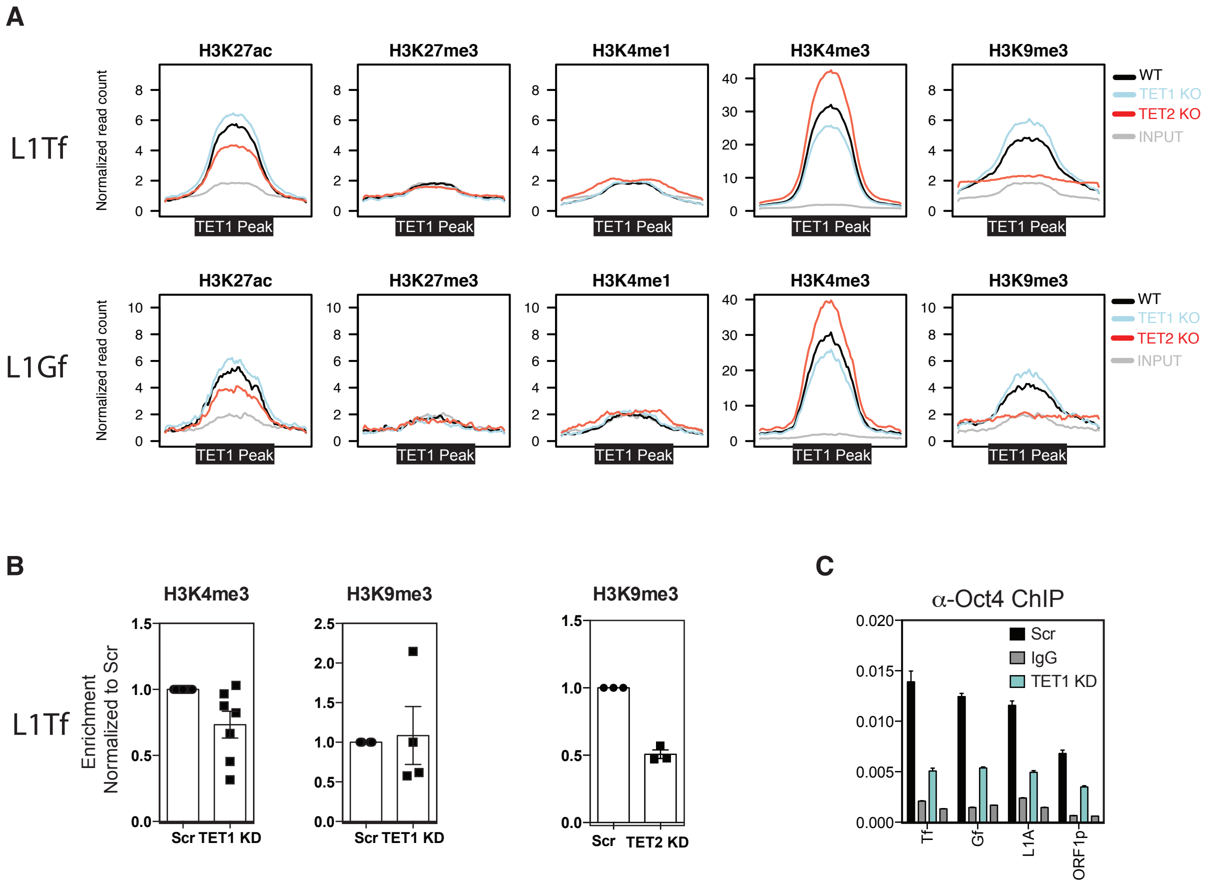

Figure S6 – ChIP data at L1s in TET-deficient ESCs. **A)** ChIP-seq profiles at TET1 peaks overlapping L1 elements in WT, *Tet1* KO or *Tet2* KO ESCs. **B)** ChIP-qPCR data for histone modifications across multiple biological replicates (n=3-7) of TET1 or TET2 shRNA experiments. **C)** OCT4 binding at the 5’ UTR of L1s is impaired upon TET1 depletion (representative replicate from n=3).

Figure S7

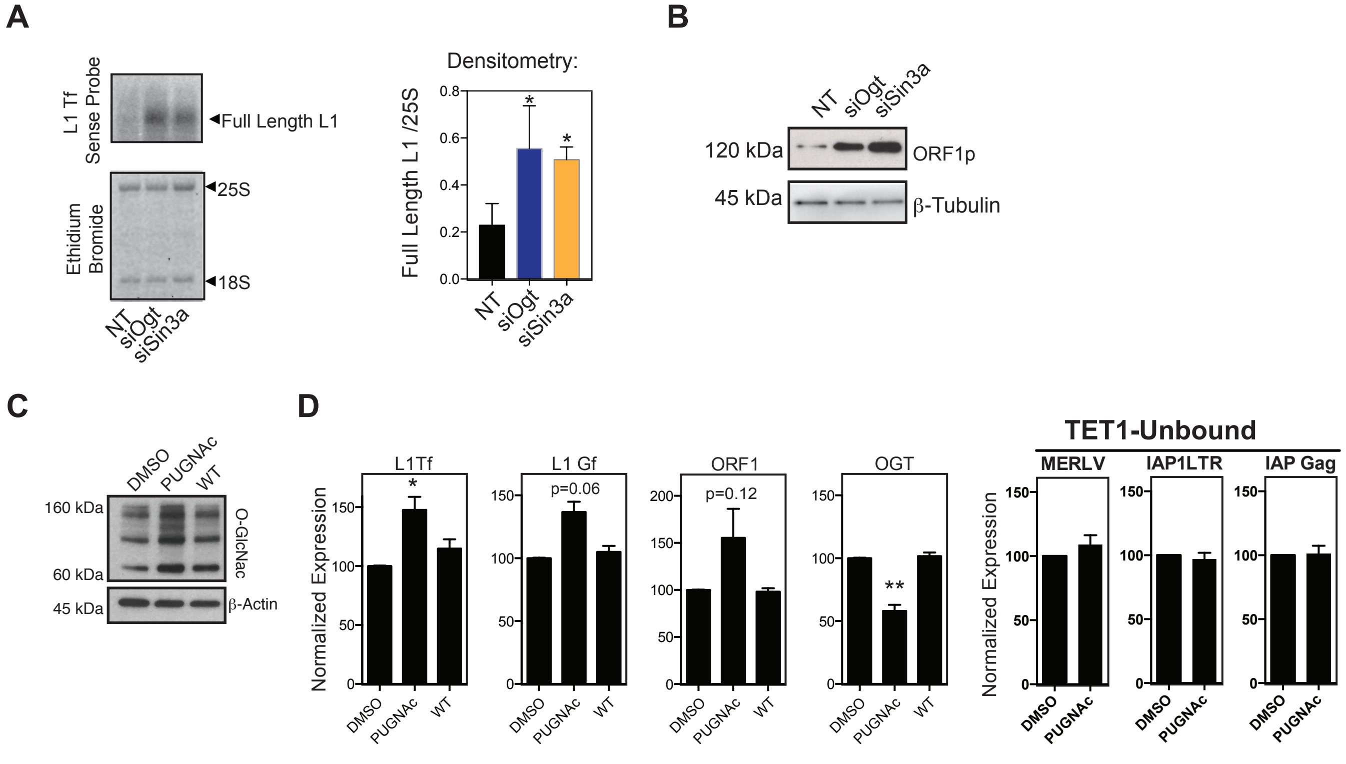

Figure S7 – Additional data on the effects of SIN3A and O-GlcNAc modulation. **A)** Northern blot data confirms that full-length L1Tf elements are upregulated upon OGT or SIN3A depletion (n=3). **B)** Western blot further shows that ORF1p protein levels are also elevated in OGT or SIN3A knockdowns. **C)** Western blot confirming that inhibition of O-GlcNAc hydrolase by PUGNAc led to raised cellular levels of O-GlcNAc. **D)** PUGNAc causes a mild increase in the RNA levels of L1s, but not of other TEs that are not TET1 targets (n=4); note that OGT levels are lower in PUGNAc-treated cells, potentially confounding the results. * p<0.05, p < 0.01, ANOVA with post-hoc test (A) or paired t-test (D).

Figure S8

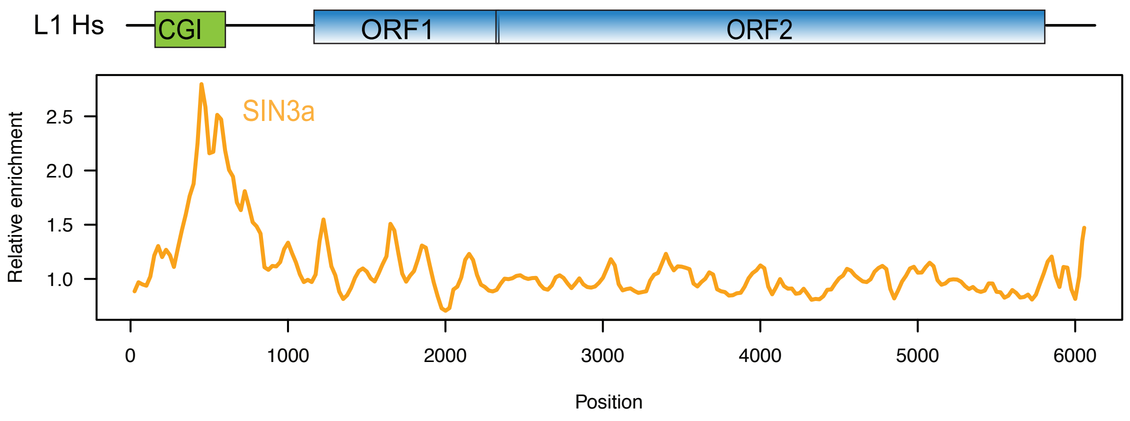

Figure S8 – SIN3A profile in human ESCs. SIN3A ChIP-seq data from human ESCs were aligned to L1.4, revealing enrichment at the 5' UTR, similar to what is seen in mouse ESCs.

Figure S9

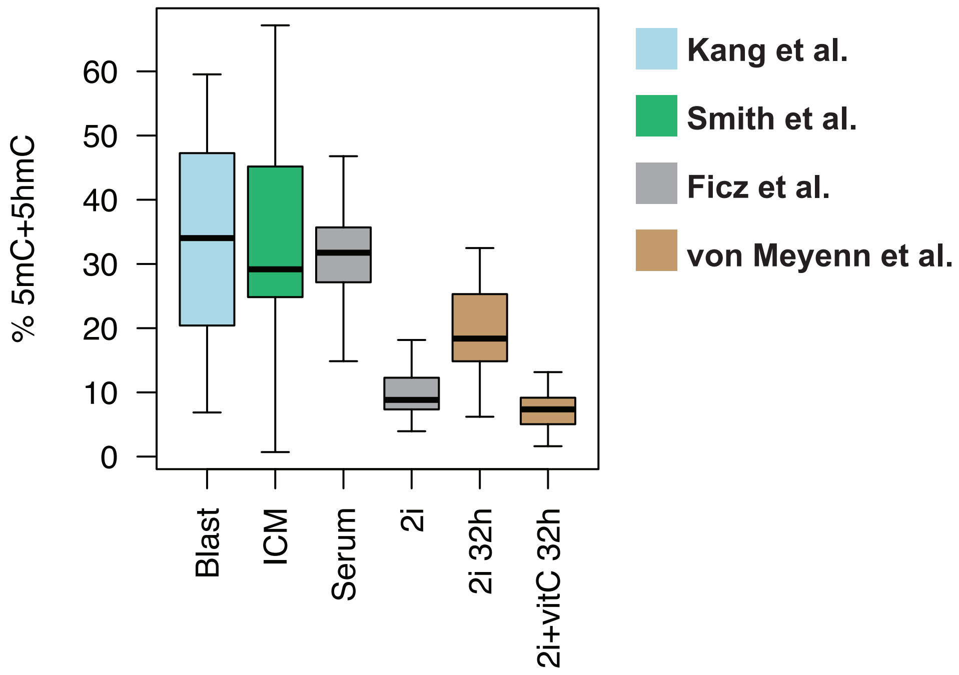

Figure S9 – L1 methylation levels *in vivo* and in ESCs grown under different conditions. Publically available BS-seq data were aligned to $L1_{Orl}$ and the methylation levels for CpGs at the 5’ UTR covered in all datasets were extracted. L1 methylation levels in blastocysts and ICM are comparable to those seen in serum-grown ESCs, whereas 2i-grown cells have substantially lower levels. In cells transitioning from serum to 2i conditions (brown boxplots), intermediate levels of L1 methylation are seen, with vitamin C driving rapid demethylation of L1s to lower levels than those seen *in vivo*.
